# A longitudinal study on the change of eating disorder-specific and nonspecific habits during weight rehabilitation in anorexia nervosa

**DOI:** 10.1016/j.ijchp.2024.100522

**Published:** 2024-11-16

**Authors:** Maria Seidel, Marie-Louis Wronski, Fabio Bernardoni, Julius Hennig, Nico Poller, Annekatrin Locke, Evelina Stender, Susanne Heckel, Veit Roessner, Stefan Ehrlich

**Affiliations:** a Division of Psychological and Social Medicine and Developmental Neurosciences, Faculty of Medicine, Technische Universität Dresden, Fetscherstr 74 01309, Dresden, Germany; b Department of Child and Adolescent Psychiatry and Psychotherapy, Carl Gustav Carus University Hospital Dresden, Fetscherstr 74 01309, Dresden, Germany; c Eating Disorder Research and Treatment Center, Department of Child and Adolescent Psychiatry, Faculty of Medicine, Technische Universität Dresden, Fetscherstr 74 01309, Dresden, Germany

**Keywords:** Anorexia nervosa, Eating disorders, Habits, Habit frequency, Ecological momentary assessment

## Abstract

**Background:**

Patients with Anorexia Nervosa (AN) are characterized by rigid behavioral patterns and habit-like routines, especially regarding food intake. It has been hypothesized that habits contribute to the maintenance of AN-related symptoms. Therefore, it is crucial to understand the role of disorder-specific and nonspecific habits during weight-restoration treatment

**Method:**

In this longitudinal study, we examined the frequency of habits using ecological momentary assessment in 44 adolescent patients with AN who were undergoing inpatient nutritional rehabilitation. All patients had two data collection periods: baseline at admission, and follow-up shortly before discharge from treatment. An age-matched healthy control group was included to assess normalization at follow-up

**Results:**

Analyses revealed a significant decrease in food-intake and hygiene-related habit frequency from baseline to follow-up. Furthermore, at follow-up habit frequency of both categories no longer differed between AN and controls. Moreover, the degree of reduction of food intake habits was predictive of weight gain at follow-up

**Conclusion:**

These findings may suggest that habitual behaviors are state factors, mainly present during the acute phase of the disorder, which advances our understanding of the habit hypothesis in AN. Changing such behaviors may be important for weight restoration, highlighting the potential value of interventions targeting habits.

## Introduction

Anorexia Nervosa (AN) is a severe mental disorder, characterized by an intense fear of gaining weight, a distorted body image, and an extreme restriction of food intake (American Psychiatric Association, 2013). These symptoms lead to a significantly low body weight and can include an excessive desire to control body shape and weight (American Psychiatric Association, 2013). Although patients with AN may at times express desires to recover, they are often unable to stop engaging in repetitive behaviors and strict eating routines that contribute to the maintenance of an extremely low weight (Steinglass & Walsh, 2016). Despite this apparent persistence, the mechanisms underlying these habit-like behaviors and their development over time, particularly in response to treatment, are not fully understood.

Habitual behaviors, conceptualized as learned, repetitive behavioral sequences, often result from a combination of stimulus-response learning and repeated actions. Over time, these actions may shift from goal-directed to stimulus-driven, becoming more automatic and less cognitively demanding (Haith & Krakauer, 2018). The habit hypothesis in AN suggests that the repetitive behaviors and strict routines seen in the disorder may result from the formation of habits rather than being driven by conscious, goal-directed actions (Uniacke et al., 2018). These behaviors, which may have been initially followed by a reward, such as positive social feedback or less anxiety, may lose their connection to the original reward over time. As a result, these habits become difficult to change, a challenge often noted in the treatment of anorexia nervosa (Davis et al., 2020). In a previous study (Seidel et al., 2022), habitual behaviors in acute AN using ecological momentary assessment (EMA) was investigated. Data on the frequency of disorder-specific (e.g., related to food preparation) and disorder-nonspecific (hygiene-related, e.g., showering, brushing teeth) habits was collected. The results demonstrated an increased frequency of habits in acute patients with AN compared to HC, even in the disorder-unspecific category. As habits are thought to allow cognitively costly actions to be performed with little effort, thereby "freeing up" resources for other demands (Marien et al., 2018; Saling & Phillips, 2007), one possible interpretation of these previous findings is that they likely reflect an adaptive mechanism to the state of energy deprivation.

Despite the growing acknowledgement of the habit hypothesis in AN, empirical research remains limited, and existing experimental studies are inconclusive (Godier et al., 2016). A few self-report studies have examined habitual behaviors in more detail, with some relating disorder-relevant habits to the severity and duration of AN (Davis et al., 2020). Although much of the research on the habit hypothesis in AN has focused on acutely ill patients, it is crucial to study the medium-term development of these habits to determine whether they are indicative of the acute state (state factors) or whether they constitute a trait marker that may have contributed to the development of symptoms (Seidel et al., 2020). However, studies have frequently used data from a single time point. As individuals undergo weight restoration, measuring how habitual behaviors change from pre- to post-treatment can provide insight into their contribution to the recovery process as well as mechanisms of treatment resistance and maintenance of the disorder.

Do AN-specific and nonspecific habits decrease or persist after weight rehabilitation treatment? Are these habits adaptive processes related to the undernourished state and/or trait factors and/or possible predictors of treatment outcome (e.g., weight gain) with potential therapeutic relevance? The aim of the current study was to extend previous findings in acutely underweight patients with AN in order to understand both eating disorder (ED)-specific (food) and nonspecific (hygiene) habit categories longitudinally. We, therefore, assessed food and hygiene habits both at an acute state and after short-term weight restoration using EMA. In case habitual behavior in AN constitutes a state marker, we hypothesized that, based on our interpretation of habitual behaviors, the need to rely on these behaviors would decrease with a healthier weight and an improved metabolic situation and reach normal levels following weight restoration. Moreover, we expected that the extent to which habitual behavior patterns (especially food-related) decrease would be predictive of weight gain. In case habits are indicative of a trait marker, we anticipated that habit frequency would not change during treatment and still be altered after short term weight-restoration.

## Method

### Participants

Data were collected from 146 individuals, 47 of whom were patients with acute AN that provided data at two measurement time points (AN_TP1 and AN_TP2), and a total of 99 healthy controls (HC) who provided data at one time point. For the current analysis, only participants with an EMA compliance rate >40 % (i.e., >40 % of situational data was available) were included, which resulted in the exclusion of three patients with AN and 17 HC. To account for potential developmental effects and to optimize comparisons between AN and HC, we implemented a pairwise age-matching algorithm (Munkres, 1957) to minimize age differences between individual AN—HC pairs. The final sample consisted of 44 female patients with acute AN (12.2–24.1 years old) and 44 female HC (12.9–24.3 years old; mean age difference=0.32 years). Patients with AN were admitted to ED programs at a German university child and adolescent psychiatry or psychosomatic medicine department, including a behaviorally oriented nutritional rehabilitation program (for details of the treatment program see Supplementary Material 1.1). They were first assessed within 96 h following treatment admission and beginning nutritional rehabilitation (AN_TP1) and again after short-term weight restoration [AN_TP2; ≥12 % Body-mass index (BMI) increase]. Although some patients reached a “normal” BMI at AN_TP2, we considered all AN_TP2 as “partially weight-restored” given that it was unclear whether they would maintain a normal weight and given the fact that the individual target weight was not always reached.

Current AN was diagnosed according to DSM-5 (American Psychiatric Association, 2013) using a modified version of the German expert form of the Structured Interview for Anorexia and Bulimia Nervosa (SIAB-EX (Fichter & Quadflieg, 2001)) and required a BMI below 17.5 kg/m^2^ (or below the 10th age percentile if younger than 15.5 years). Information about comorbid diagnoses in patients with AN was obtained from the SIAB-EX and medical records and confirmed by an expert clinician. To be included in the HC group, participants had to be of normal weight and eumenorrheic. Normal weight was defined as BMI above 18.5 kg/m^2^ (if older than 18 years)/above the 10th age percentile (if younger than 18 years) and BMI below 30 kg/m^2^ (if older than 18 years)/below the 95th age percentile (if younger than 18 years). History of any ED or other psychiatric disorder was an exclusion criterion for HC. HC were recruited through advertisement among middle school, high school, and university students. Inclusion and exclusion criteria as well as possible confounding variables were obtained using semi-structured research interviews, the SIAB-EX (Fichter & Quadflieg, 2001), and the Mini-International Neuropsychiatric Interview (M.I.N.I. (Sheehan et al., 1998, 2010)). For further inclusion/exclusion criteria see Supplementary Material 1.2.

Study data were collected and managed using secure, web-based research electronic data capture tools REDCap (Harris et al., 2009). Our study was approved by the local Institutional Review Board, and all participants (and, if underage, their guardians) gave written informed consent.

### Clinical measures

To complement the information obtained with the clinical interviews, we assessed ED-specific psychopathology using the Eating Disorder Inventory-2 (EDI-2 (Thiel et al., 1997)), depressive symptoms using the Beck Depression Inventory-II (BDI-II (Hautzinger et al., 2009)), and obsessive-compulsive traits using the “Zwangsinventar für Kinder und Jugendliche” (ZWIK-S (Goletz & Döpfner, 2007, 2011)), a validated German self-report questionnaire to assess obsessive-compulsive symptoms. Please consult Supplementary Material 1.3 for details. BMI and BMI-standard deviation score (BMI-SDS (Hemmelmann et al., 2010; Kromeyer-Hauschild et al., 2001) were measured in HC and in AN at both time points one day before starting the EMA assessment. In a subset of the current study sample (*n* = 37 AN_TP1; *n* = 39 AN_TP2; *n* = 43 HC), venous blood for leptin analysis was collected after an overnight fast (see Supplementary Material 1.4).

### EMA measures & materials

The current data collection used the same measures and followed the same study protocol as a previous study (Seidel et al., 2022). Participants were provided with a study smartphone. The app-based questionnaire was designed via an online platform (MovisensXS, Karlsruhe, Germany). When participants received the study smartphone, they were briefed on what types of behaviors constitute habits (details see below). The EMA asked participants at each prompt whether they had carried out habits within the last 60 min, and if so, in which of several categories the activities could be classified (e.g., food intake, hygiene, or public transport; see Supplementary Material 1.3). Given that most patients with AN in the current study were inpatients, which restricted daily routine, e.g., related to activities in traffic or meal preparation, the focus of the current study was on habits that occurred in one ED-specific (food intake) and in one ED-unspecific (hygiene) category only. Both categories were assumed to be compatible with daily life while participating in a behaviorally oriented intensive treatment program and comparable to the data assessed during daily life of the HC group. At each prompt, participants could enter up to five different habits.

### Procedure

Participants were initially screened, weighed, interviewed and, as previous studies (Fürtjes et al., 2019; Seidel et al., 2022), received detailed instructions on how to handle the study smartphone, the EMA app, and the content of the questionnaires. At the first assessment, each participant completed a tutorial, during which they received five example descriptions of habitual behavior sequences of different categories. Furthermore, participants were instructed to answer the questionnaires as soon as the alarm appeared but were given an additional 30 min after the prompt if they were unable to reply (e.g., during class or work, during mealtimes or therapy sessions). As in previous EMA studies (Fürtjes et al., 2019; Seidel et al., 2022), sampling started the day after screening and lasted for a period of 7 days via the signal-contingent assessment method: alarms occurred at 8 semi-random times during a 14 hour period that was adapted for each individual to suit different daily routines. Compensation was provided at the end of the study in accordance with compliance rates.

### Statistical analysis

Since our research design (EMA) produced nested data (Hox, 2000), i.e., individual prompts were nested within participants, we conducted several hierarchical generalized linear models (HGLM) for binomial outcome variables with the statistical software R (Version 4.2.1) using the lme4 package (Bates et al., 2015). We specified the occurrence of a habit as “1” (for either food intake [Model 1] or hygiene [Model 2]) or no habit as “0” for each prompt, which constituted the primary outcome measure of interest. To account for the hierarchical structure of the data, time point (AN_TP1, AN_TP2) and participant were included as random effects (prompts were nested within time points, which were nested within participants). For a detailed overview of the analysis flow, see Supplementary Material Figure S1. Initial analysis included validation of the chosen model structure (3-level random intercept and random slope model) against 2-level and 4-level model structures (Supplementary Material Table S1). Main fixed effects were time point (baseline: 0 for AN_TP1 and HC; follow-up: 1 for AN_TP2; factorized) and group (patient: 0 for AN_TP1 and AN_TP2; control: 1 for HC; factorized). For both models, age of participant and compliance rate were also entered as control variables. Additionally, prompt count, representing the study progress, was entered at the situation level to account for possible effects of study duration. Cross-sectional differences (AN_TP1 vs. HC) to validate the original findings (Seidel et al., 2022) and longitudinal changes in habitual behavior (AN_TP1 vs. AN_TP2) were investigated applying contrast analyses. Models for food intake (1) and hygiene (2) habits were estimated separately. Benjamini-Hochberg false discovery rate (FDR) correction of p-values was performed across all assessed contrasts for each category to account for multiple testing. To estimate how changes in habit occurrence were related to individual weight gain, we estimated additional supplementary analyses replacing the fixed effect of time point (factor with levels AN_TP1 and AN_TP2) with change in BMI-SDS from AN_TP1 to AN_TP2 (continuous variable Delta-BMI-SDS).

In a next step (sensitivity analysis), as previously done in a cross-sectional study (Seidel et al., 2022), a ratio representing habit frequency was calculated for each category separately. The ratio was calculated as the number of times participants reported a habit that could be assigned to either the food intake (Frequency-food) or hygiene (Frequency-hygiene) category, divided by the total number of answered prompts (including all categories (see Supplementary Material 1.3) as well as prompts where participants responded that they did not notice any habitual behavior). We validated longitudinal HGLM findings for changes in habits by testing the computed frequency measures (Frequency-food, Frequency-hygiene) in AN_TP1 vs. AN_TP2 with the help of dependent sample *t*-tests. Additionally, we calculated a change score in the frequency measure for each category (Delta-food; Delta-hygiene) in patients with AN by subtracting the frequency of AN_TP1 from AN_TP2. To investigate whether these changes in habit frequency would predict weight gain from AN_TP1 to AN_TP2, we conducted two linear regression models with Delta-BMI-SDS as outcome and Delta-food and Frequency-food at AN_TP1 (Model A) or Delta-hygiene and Frequency-hygiene at AN_TP1 (Model B) as well as BMI-SDS at AN_TP1 and age at AN_TP1 as predictors for both models. Further, as part of an exploratory analysis, Pearson's correlations were calculated between our habit scores (Frequency-food and Frequency-hygiene) and duration of illness as well as compulsive symptoms (ZWIK).

The final step of our analysis involved evaluating the degree of normalization after weight restoration treatment by a) looking at the contrast AN_TP2 vs. HC in our main HGLM models (Model 1 & 2) and b) evaluating the strength of evidence supporting the null hypothesis of no group differences between patients at AN_TP2 and HC using Bayesian-framework analyses (in case of absent group differences in the frequentist approach). Thus, for nonsignificant contrasts between AN_TP2 and HC, HGLM models within the Bayesian framework (Muth et al., 2018) were estimated. For both models, the respective Bayes Factors (BF_01_) were calculated indicating the amount of evidence in favor of the null hypothesis (threshold: BF_01_ >3 for moderate evidence, BF_01_>10 for strong evidence).

## Results

### Descriptive and clinical data

Table 1 summarizes the results of group comparisons in demographic and clinical variables. Patients spent on average 98.2 days in treatment (SD=29.19). During this time, the mean percent BMI increase was 32.94 % (SD=10.56, range=18.58 %−65.48 %). As expected, BMI-SDS, leptin, and cognitive symptoms improved significantly from AN_TP1 to AN_TP2; however, some residual symptoms were still present (comparing AN_TP2 and HC).

**Table 1:** Results of group comparisons of descriptive data using dependent sample and independent sample *t*-tests, reporting mean (M), standard deviation (SD), t values and statistical significance (p). Frequency-food/hygiene is the total amount of reported food/hygiene habits divided by the total amount of answered prompts over the duration of seven days. *N* = 12 (27.27 %) of patients with AN were of the binge-eating/purging subtype. AN_TP1=patients with acute anorexia nervosa, AN_TP2=patients with AN after weight restoration program, BMI=body mass index, BMI-SDS=body mass index standard deviation score, BDI-II=Beck Depression Inventory II, compliance=percentage of answered prompts with a possible total of 56, DOI=duration of illness in months, EDI-2=Eating Disorder Inventory 2, HC=healthy control, Log_10_Leptin=log _10_ leptin in ng/ml with imputed values for leptin below detection limit of the assay (see Supplementary Material 1.5. Statistical Analysis), ZWIK=“Zwangsinventar für Kinder und Jugendliche” total score and washing compulsion (WC subscale).

	AN_TP1		AN_TP2		HC		AN_TP1 vs. AN_TP2		AN_TP2 vs. HC	
	*M*	*SD*	*M*	*SD*	*M*	*SD*	*t*	*p*	*t*	*p*
Age	15.44	1.47	15.72	1.57	15.65	1.70	−19.64	**<0.001**	0.21	0.835
BMI-SDS	−3.28	1.07	−0.60	0.49	0.01	0.65	−19.72	**<0.001**	−5.00	**<0.001**
Log_10_Leptin	1.40	2.16	13.03	6.72	11.89	8.15	−11.89	**<0.001**	0.69	0.491
EDI-2	217.38	40.80	222.66	45.15	138.36	29.11	−0.80	0.431	10.15	**<0.001**
BDI-II	25.79	10.14	21.38	12.07	5.95	5.46	2.44	**0.019**	7.57	**<0.001**
DOI	9.53	8.04								
ZWIK-total	49.96	18.14	50.99	20.52	42.08	12.01	−1.02	0.315	2.40	**0.020**
ZWIK-WC	14.65	5.45	15.46	6.93	12.74	5.54	−1.07	0.292	2.00	**0.049**
Compliance (%)	0.85	0.14	0.85	0.16	0.75	0.17	0.26	0.800	2.74	**0.008**

As in previous studies (Fürtjes et al., 2019; Seidel et al., 2022), compliance in EMA was generally good in both groups, but significantly higher in AN than HC, with patients answering on average 85 % of prompts (at each time point AN_TP1 and AN_TP2) compared to 75 % in HC.

### EMA data

**Fig. 1:**
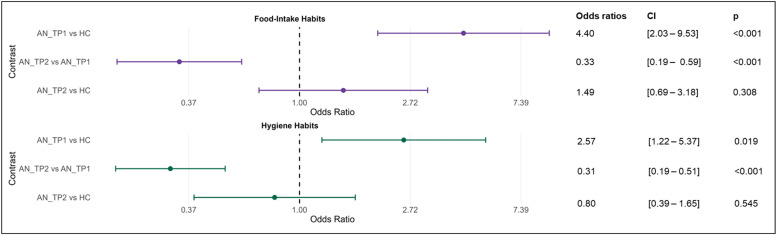
Odds ratio for each contrast according to the output of the hierarchical general linear models (Model 1 outcome: Food intake habits (top), Model 2 outcome: Hygiene habits (bottom)). An odds ratio >1indicates higher odds for habit occurrence in the first group compared to the second group, an odds ratio <1 indicates lower odds for habit occurrence in the first group compared to the second group. AN_TP1=acute anorexia nervosa patients at admission; AN_TP2=anorexia nervosa patients at follow-up; HC=Healthy control; CI=Confidence Interval.

Altogether, participants provided 5915 data points, during which either a food intake (*n* = 1805), hygiene (*n* = 1846), or no (*n* = 2264) habit was reported (for examples of specific types of habits see Supplementary Material 2.1). Results of the initial multilevel analysis examining cross-sectional differences (contrast AN_TP1 vs. HC) were consistent to previous findings in acute AN, showing a significantly higher odds of reporting food intake as well as hygiene habits in AN patients (Table 2 and Figure 1; in this contrast an odds ratio >1 (OR=4.04) indicates that the odds of habit occurrence in AN patients (first group) are higher compared to HC (second group)). Looking at the longitudinal contrast (AN_TP1 vs. AN_TP2), results indicated a significant decrease in the odds of habit occurrence for both outcomes (food intake [Model 1] and hygiene [Model 2], see Table 2 and Fig. 1) from time point AN_TP1 to AN_TP2. In this contrast, the odds ratio below 1 indicates that the odds of habit occurrence at AN_TP2 are lower compared to AN_TP1.These longitudinal differences were confirmed using paired *t*-tests showing that, over the course of the inpatient treatment, Frequency-food and Frequency-hygiene decreased significantly (sensitivity analysis, Supplementary Material Table S2). Supplementary analyses, replacing time point (AN_TP1, AN_TP2) with Delta-BMI-SDS in Model 1 and 2 revealed that change in BMI-SDS was an even better predictor than time point to explain variance in habit occurrence of both outcomes at AN_TP2 (Supplementary Material Table S3).

**Table 2:** Results of the two hierarchical generalized linear models, as implemented in R statistical software, with food habits (Model 1) and hygiene habits (Model 2) as outcomes. Results report odds ratios, confidence intervals, and p values. The factor group was coded 0 for AN_TP1 and AN_TP2 and 1 for HC, therefore the reference group is the patient group and odds ratios of a habit occurring are displayed for HC relative to AN. An odds ratio <1 represents a decreased likelihood in relation to the comparison group, and odds ratio >1 an increased likelihood.

Model 1: Food intake				Model 2: Hygiene		
*Predictors*	*Odds Ratios*	*95 % CI*	*p*	*Odds Ratios*	*95 % CI*	*p*
(Intercept)	36.78	1.40 – 967.93	**0.031**	20.26	0.79 – 522.35	0.07
Group [HC]	0.23	0.10 – 0.49	**<0.001**	0.39	0.19 – 0.82	**0.013**
Time point [TP2]	0.34	0.19 – 0.59	**<0.001**	0.31	0.19 – 0.51	**<0.001**
Age	0.92	0.76 – 1.13	0.437	0.89	0.73 – 1.09	0.268
Prompt count	0.99	0.98 – 0.99	**<0.001**	0.98	0.98 – 0.99	**<0.001**
Compliance	0.22	0.03 – 1.56	0.129	0.82	0.13 – 5.23	0.834
**Random Effects**						
σ^2^	3.29			3.29		
τ_00__participant_id:tp_	1.5			1.09		
τ_00__participant_id_	0.77			0.89		
ICC	0.45			0.44		
Observations	4069			4110		
Marginal R^2^ / Conditional R^2^	0.08 / 0.49			0.06 / 0.48

**Fig. 2:**
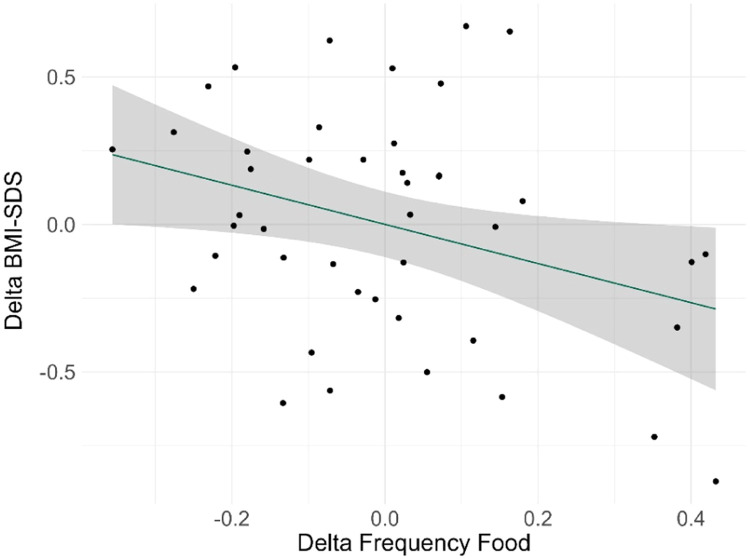
Association between residualized Delta-BMI-SDS and residualized Delta-food (change in food intake habits). Linear regression accounts for the effects of Frequency-food, Age, and BMI-SDS at AN_TP1. Negative association indicates that a greater reduction in habits from AN_TP1 to AN_TP2 is associated with higher weight gain.

Linear regressions revealed that Frequency-food at AN_TP1 and Delta-food, but not Frequency-hygiene at AN_TP1 or Delta-hygiene explained significant variance in weight gain at AN_TP2 (Delta-BMI-SDS). The lower the original habit level and the more food intake habits decreased during treatment, the more weight was gained from AN_TP1 to AN_TP2 (Table 3, Fig. 2). Given plotting the data yielded that the association between Delta-food and Delta-BMI-SDS might have been driven by one outlier, analyses were repeated using robust linear regression (MASS package in R) and excluding the extreme value. Neither approach changed the significance of the results (Supplementary Material Table S4A/S4B). No such associations were found for Frequency-hygiene at AN_TP1 or Delta-hygiene (see Supplementary Material Table S5). In an exploratory analysis we did not observe any significant correlations between Delta-food/Delta-hygiene and duration of illness (in months) (Food intake: *r* = 0.05, p_uncorrected_=0.8; Hygiene: *r* = 0.11, p_uncorrected_=0.5) or Delta-Zwik (food-intake: *r* = 0.08, p_uncorrected_=0.8; hygiene: *r* = 0.25, p_uncorrected_=0.13).

**Table 3:** Linear regression predicting Delta BMI-SDS with food intake habits frequency (change and baseline), age and BMI-SDS at baseline. CI=Confidence interval. Significant p values are highlighted in bold.

*Predictors*	*Estimate*	*95 % CI*	*p*
(Intercept)	1.37	0.11 – 2.63	**0.034**
Delta-food	−0.84	−1.39 – −0.28	**0.004**
Frequency-food at AN_TP1	−0.66	−1.27 – −0.06	**0.033**
Age	−0.06	−0.14 – 0.02	0.13
BMI-SDS at AN_TP1	−0.74	−0.86 – −0.62	**<0.001**
Observations	44		
R^2^ / R^2^ adjusted	0.839 / 0.823

Contrast analysis comparing AN_TP2 and HC did not reveal any significant difference for the occurrence of a habit of either category in our HGLMs (Table 2, Fig. 1). BF_01_ for food intake was 8.8 indicating moderate evidence for the null hypothesis (no group differences), BF_01_ for hygiene 14.87, indicating strong evidence for the null hypothesis indicating a normalization in habit frequency after short-term weight restoration.

## Discussion

The goal of the current study was to investigate the change of food intake- and hygiene-related habitual behaviors before and after successful weight restoration treatment in a sample of adolescent patients with AN. We found a significant decrease in habit occurrence from the initial time point (AN_TP1) to the follow-up assessment (AN_TP2) for both food intake and hygiene habits. The results indicate that habit frequencies decrease and approach normal levels (as compared with HC) over the course of short-term weight restoration suggesting a state-related mechanism. Moreover, a lower frequency of food intake habits at AN_TP1 as well as higher reduction of habits over time was found to be predictive of higher weight gain during treatment. A speculative interpretation could be that a successful reduction of these repetitive behaviors and a generally low tendency to display them might facilitate weight gain and an overall more successful inpatient treatment.

As previously reported and validated with the current analyses, patients with AN showed increased habit-frequency in both categories at AN_TP1. Findings of higher habit frequency during the acute state compared to HC have been suggested to serve as an adaptive function by allowing complex, cognitively costly actions to be performed with little deliberation, thereby “freeing up” processing capacities for higher-order strategic tasks (Mendelsohn, 2019; Wood, 2017). Consequently, the efficiency of habits appears particularly relevant under conditions of heavy load, such as exhaustion or pressure (Verplanken & Orbell, 2003). Taken together, severe cognitive and endocrine stresses during the undernourished state of acute AN may lead to operating in a “habitual” mode, allowing patients with AN to function in a more energy-efficient way (Park et al., 2017; Smeets et al., 2019). The current results seem to support this habit-efficiency hypothesis. At the end of treatment patients not only have an improved weight and metabolic state (including a significant change in leptin) but also fewer psychological symptoms e.g. depressive symptoms. Consequently, the need for the intense use of habits might be lower after (partial) weight-recovery. The pattern of results supports the assumption that, already with short-term weight stabilization, patients with former AN show a normalized frequency of habitual behavior. Thus, these findings strengthen the notion that the reliance on habitual behavior patterns in patients with AN can be considered a state-related factor. As such, increased habit reliance is only present during the acute phase, probably reflecting an adaptation to the state of undernutrition, rather than an underlying trait (e.g., a general tendency to develop habits more easily or faster than HC), which could have contributed to the development of the disorder (Uniacke et al., 2018). However, in obsessive compulsive disorder (OCD), which is genetically related to AN (Yilmaz et al., 2020), an experimental study examining model-based (goal-directed) and model-free (habitual) learning reported that, even after Cognitive Behavioral Therapy treatment, deficits in model-based learning persisted. The findings may indicate that the shift toward model-free behavior in OCD might be more persistent (Wheaton et al., 2019). Our findings may serve as an important specification of the habit hypothesis in AN. However, further longitudinal studies as well as studies in long-term recovered patients will be necessary for validation.

Of the two habit categories, only the disorder-specific (food intake) habits at AN_TP1 and the corresponding change to AN_TP2 were predictive of weight gain between the two time points. This association implies that targeting disorder-specific habits in the treatment of AN might be a useful addition to standard therapeutic approaches. To give an example, the REACH+ program, as outlined in a recent study (Steinglass et al., 2022), presents a promising approach: the program emphasizes real-world practice so that patients are encouraged to confront and alter habitual behaviors in their daily environments. Through repeated exposures and the use of tailored interventions like cue disruption and reinforcement strategies, the program aims to weaken the hold of old habits and replace them with more adaptive behaviors.

Of note, as an alternative to the habit theory of AN, certain elements of the eating behaviors that individuals with AN engage in repeatedly could also be interpreted as eating rituals (Calugi et al., 2019). In contrast to habitual actions, ritualistic behaviors tend to be purposeful and directed towards specific goals (Tonna et al., 2019). They are thought to help individuals regain a sense of control (Lloyd et al., 2021), which is consistent with our findings of increased self-control in AN (King et al., 2019; Pauligk et al., 2021). Although habitual and ritualistic behaviors may appear similar on a surface level, they may represent two distinct phenomena, each with its own underlying processes (Tonna et al., 2019). Consequently, we encourage future EMA studies investigating habitual behavior in AN to distinguish between these two behavior patterns and elucidate their meaning for ED symptoms.

Several limitations of this study warrant discussion. First, the inpatient treatment setting may introduce biases regarding the opportunities for certain habitual behaviors, as patients are under close supervision and control. While we acknowledge that the influence of the inpatient setting cannot be excluded, it likely had less impact in the longitudinal approach, as the setting did not change significantly between assessments. Nonetheless, more daily life data from AN outpatients, including larger sample sizes, are needed to support the conclusion. A second limitation might be that we did not monitor momentary symptom severity as part of our EMA data collection. Consequently, we are currently unable to determine whether habits at the micro-level exacerbate or alleviate AN-related symptomatology within very short time windows. Interestingly, we did not observe statistically significant changes in eating disorder symptoms between AN_TP1 and AN_TP2. As the primary focus of the treatment program in our study was on achieving medically necessary weight restoration, it is important to acknowledge that this process can be psychologically challenging for patients. Emotional and cognitive responses to weight gain may (in some patients) temporarily exacerbate or maintain some of the eating disorder symptoms. Symptomatic improvement often requires more time and is typically seen after sustained weight recovery. Another challenge, which also extends to other studies employing similar methodologies, lies in the difficulty of distinguishing between disorder-specific habits and symptoms as well as the overlap with similar but distinct constructs such as compulsive behaviors (Gillan et al., 2014) . To address the latter point, we demonstrated that our habit measures were distinct from compulsive behaviors. Future studies could explore specific types of these habits in greater detail. Finally, there are inherent challenges in assessing automatic behaviors using self-report measures. Despite the advantages of EMA in capturing real-time data, reliance on self-report may still be limited in accurately reflecting the deeply ingrained and unconscious nature of habits.

## Conclusion

Taken together, the current study suggests that habitual behaviors related to food intake and hygiene are state factors, mainly present during the acutely underweight state of AN. This finding advances our understanding of the habit hypothesis in AN. The observed longitudinal reductions in habitual behaviors in AN, and even more so their ability to explain weight gain during treatment, may be crucial for devising successful treatment programs. This insight underscores the potential benefits of therapeutic interventions like habit reversal training.

## Declaration of competing interest

The authors declare that they have no known competing financial interests or personal relationships that could have appeared to influence the work reported in this paper.

## Appendix Supplementary materials

Image, application 1
