# Supplementary material for: A longitudinal study on the change of eating disorder-specific and nonspecific habits during weight rehabilitation in anorexia nervosa
Source: Int J Clin Health Psychol. 2024 Nov 16;24(4):100522. doi: 10.1016/j.ijchp.2024.100522 (PMC11612769; doi:10.1016/j.ijchp.2024.100522)
Supplement: Supplementary file 1 [file mmc1.docx]

**Supplementary Material: A longitudinal study on the change of eating disorder-specific and nonspecific habits during weight rehabilitation in anorexia nervosa**

1. Methods
   1. Treatment program

During inpatient treatment, patients were enrolled in a comprehensive multimodal psychiatric and psychotherapeutic treatment program. Central elements of the treatment program were an operant behavioral program to achieve weight gain (>700g per week) including a meal-plan and supervised food intake. Furthermore, cognitive-behavioral treatment in individual, family, and group therapy sessions was provided. This was supplemented by individual as well as group-based body-focused psychotherapy including exposure therapy, as well as a relapse prevention module and reintegration into community activities in the last third of the treatment program. Habitual behavior was not explicitly targeted during supervision or in the behavior interventions.

- 1. Participants

Clinical data were also collected from all participants using our own semi-structured research interview which includes several specific questions to assess menstruation history, weight history, general medical and medication history, family psychiatric history, ethnicity, smoking status, and socioeconomic factors (e.g., educational level, occupation, family status, current living situation). Participants of both the acute anorexia nervosa (AN) and healthy control (HC) groups at the focus of the current analyses were excluded if they had a history of organic brain syndrome, schizophrenia, substance dependence, psychosis not otherwise specified bipolar disorder, bulimia nervosa or binge-eating disorder. Further exclusion criteria were: IQ<85, psychotropic medication within 6 weeks prior to the study, current substance abuse, inflammatory, neurologic or metabolic illness, chronic medical or neurological illness that could affect appetite, eating behavior, or body weight, clinically relevant anemia, pregnancy or breast feeding. Of the n=44 AN patients, TP_1 datasets of n=42 (95.46%) were included in the analysis of our previous manuscript reporting on differences in habit-frequency between acute patients and HC.

- 1. Clinical and EMA measures

Participants were instructed to focus on automated, routine behaviors that are performed with little to no conscious thought, such as brushing teeth or locking a door. They were given examples to illustrate how habits typically consist of a sequence of actions carried out in the same way each time. The instructions emphasized that habits are often triggered by specific contextual conditions in daily life. Participants were guided through a tutorial that described five different situations, encouraging them to identify and log up to five habits that occurred in the last hour during each prompt. The examples provided were intended to help participants recognize the types of behaviors the study was interested in tracking.

Further examples were: Eating: 1) Always eat the vegetables first. 2) Always arrange food in the same way (vegetables on the left side of the plate, meat on the right side of the plate). 3) Count when chewing (chew at least 5 times before swallowing). Hygiene: 1) Brushing teeth (first the upper jaw, then the lower jaw; first left, then right.). 2) Washing hair (put shampoo in the left hand with the right hand, then distribute the shampoo on the head with both hands.). 3) Washing hands (put soap in the left hand with the right hand, then open the tap with the right hand.).

During each prompt, participants were asked whether, in the last 60 minutes, they had carried out a habit, and if yes in which of eight categories the activity, which was labelled as a habit, could be categorized. Categories were “food preparation”, “eating”, “hygiene”, “styling”, “control”, “transport”, “sport”, or “miscellaneous”.

To assess obsessive-compulsive traits we used the German self-report questionnaire “Zwangsinventar für Kinder und Jugendliche” (ZWIK-S (Goletz & Döpfner, 2007)). The ZWIK-S is a multidimensional self-report questionnaire and serves the assessment of obsessive-compulsive symptoms in children and adolescents. The 36 items of the questionnaire are grouped into four subscales (contamination thoughts and washing compulsions; control and repetition compulsions; compulsive thoughts regarding harming or injuring others or oneself; counting and questioning compulsions) as well as a total (summary) score. For the ZWIK-S, high internal consistency for the total score (Cronbach's alpha=.92) and sufficient to good internal consistencies for the subscales (Cronbach's alpha=.77-.86) were reported (Goletz & Döpfner, 2011).

- 1. Blood samples

In a subset of the current study sample (n=37 AN_TP1, n=39 AN_TP2 and n=43 HC), venous blood for leptin analysis was collected into vacutainer tubes between 7 and 9 a.m. following an overnight fast within 96h of starting the rehabilitation program. The blood samples obtained were immediately processed as follows: addition of the serine protease inhibitor aprotinin, centrifugation (at ϑ = 5 °C and a = 2,500 x g for 15 min), aliquotation into Eppendorf Tubes, storage at ϑ = -80 °C until laboratory analysis. Plasma leptin concentrations were measured using a commercially available enzyme-linked immunosorbent assay (ELISA; BioVendor Research and Diagnostic Products, Brno/Czech Republic) with intra- and inter-assay variation coefficients of <10%. Leptin served as an indicator variable for neuroendocrine alterations and nutritional status in AN (Hebebrand et al., 2007).

1.5. Statistical Analyses

Leptin concentrations below the lower limit of detection of the applied assay (LOD=0.20ng/mL) within the AN sample (n=16 (36.36%)) were imputed using a quantile regression multiple imputation approach for left-censored missing data (QRILC). QRILC performs random draws from a truncated distribution with parameters estimated using quantile regression (derived from the distribution of existing leptin concentrations within detection range; please note that leptin values were log10-transformed and that no further covariates were introduced in the imputation model). QRILC was conducted in R with the help of package "imputeLCMD" (Lazar, 2015). A Gibbs sampler based approach (Wei et al., 2018) with n=100 iterative draws per value from the specified truncated distribution was then used to update the initialized values from QRILC and to ensure that the imputed leptin values were positive (on the original scale, i.e., >0) and below LOD.

1. **Results**

2.1. Habit description

Food-intake habits of AN and HC were quite different. Patients exhibited highly specific habitualized eating behaviors. For example, some AN participants reported eating in a specific order, such as eating the edges of a sandwich first, then eating the bread from the outside to the center while holding it in their right hand. One patient described habits such as cutting a pear into exactly three pieces before eating, or carefully sorting their food before eating it. Another patient described chewing each bite of her evening meal between 15 and 20 times, or starting with eating the cheese or sausage that protruded from the bread.

In contrast, HC reported less rigid habits, often involving more common or general practices around eating. For example, one participant mentioned peeling and eating a carrot by first cutting off the ends and then peeling it with a knife. Another described skimming the milk foam from her coffee before drinking the rest, while another mentioned breaking off pieces of a chocolate bar and eating them slowly after carefully unwrapping it.

**Supplementary Figures and Tables**

**Figure S1: Analysis Flowchart**


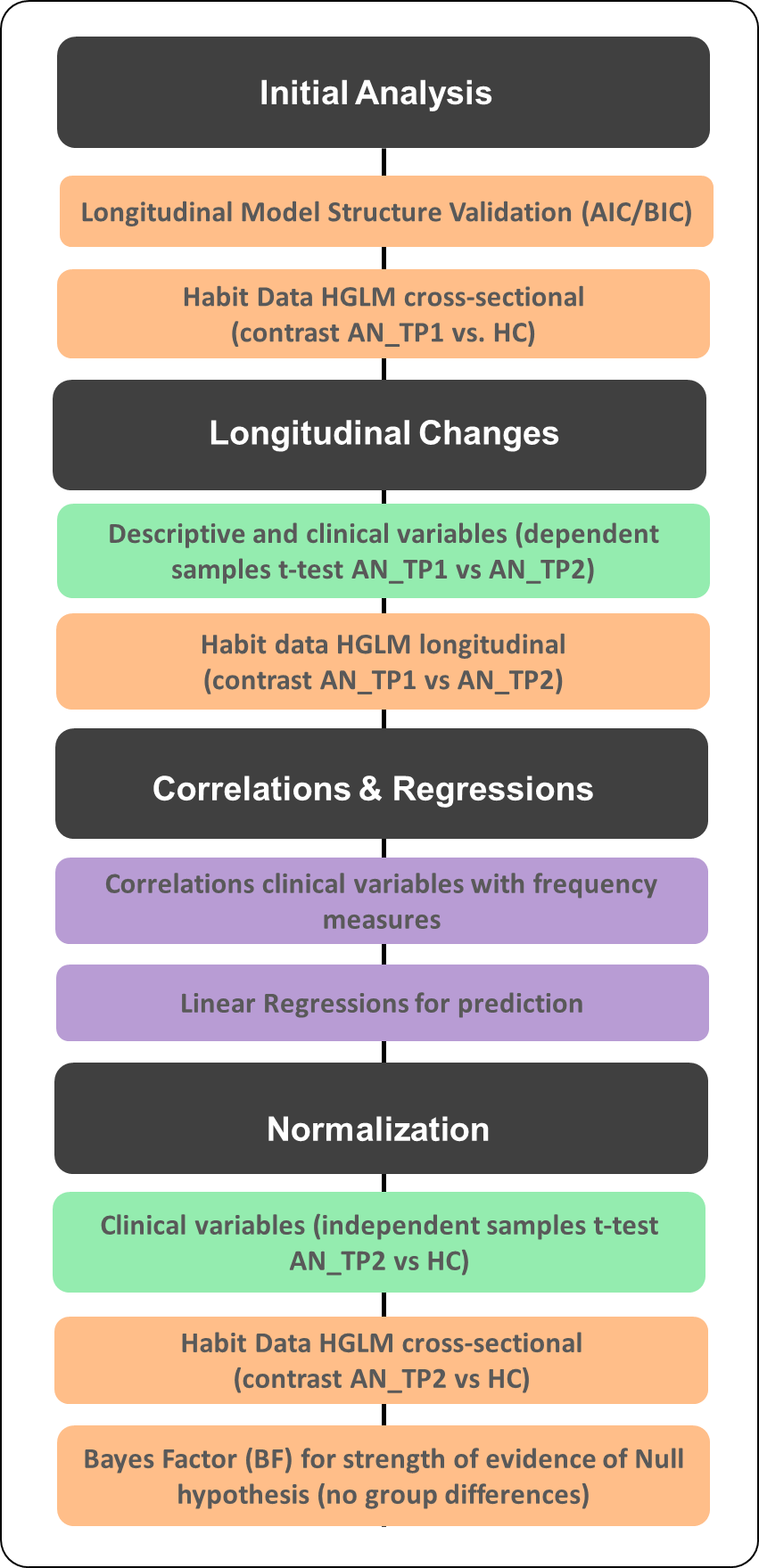


| **Model** | **Random intercept** | **Random slope** | **AIC** | **BIC** |
| --- | --- | --- | --- | --- |
| 2 Level | Participant | / | 6364 | 6411 |
| 2 Level | Participant | Participant/Prompt | 6354 | 6414 |
| 3 Level | Participant, TP | / | 6167 | 6221 |
| 3 Level | Participant, TP | Participant/Prompt | 6154 | 6221 |
| 4 Level | Participant, TP, day | / | 6169 | 6229 |
| 4 Level | Participant, TP, day | Participant/Prompt | 6156 | 6229 |

**Table S1: Model validation**

**Notes:** AIC and BIC of models with different hierarchical structures. Decision for **Model with 3 levels** with random intercept and random slope was made based on low AIC as well as low BIC. AIC=Akaike information criterion; BIC=Bayesian information criterion.

|  | **AN_TP1** |  | **AN_TP2** | |  | **AN_TP1**  **vs.**  **AN_TP2** | |
| --- | --- | --- | --- | --- | --- | --- | --- |
|  | ***M*** | ***SD*** | ***M*** | ***SD*** | | ***t*** | ***p*** |
| Frequency-Food | 0.41 | 0.28 | 0.31 | 0.23 | | 2.23 | 0.031 |
| Frequency-Hygiene | 0.40 | 0.28 | 0.27 | 0.23 | | 3.20 | 0.003 |

**Table S2: Cross-sectional and longitudinal changes in frequency measures (averaged data)**

| **Table S3: Longitudinal Changes during Weight Restoration** | | | |  | | |  |  |
| --- | --- | --- | --- | --- | --- | --- | --- | --- |
| **Model 1b: Food intake** | | | | **Model 2b: Hygiene** | | |  |  |
| *Predictors* | *Odds Ratios* | *95% CI* | *p* | *Odds Ratios* | *95% CI* | *p* | |  |
| (Intercept) | 37.46 | 1.41 – 996.64 | **0.03** | 21.57 | 0.79 – 588.03 | 0.069 | |  |
| Group | 0.21 | 0.10 – 0.45 | **<0.001** | 0.37 | 0.18 – 0.79 | **0.01** | |  |
| Delta BMI-SDS | 0.64 | 0.53 – 0.77 | **<0.001** | 0.65 | 0.55 – 0.77 | **<0.001** | |  |
| Age | 0.94 | 0.77 – 1.15 | 0.535 | 0.91 | 0.74 – 1.12 | 0.352 | |  |
| Prompt count | 0.99 | 0.98 – 0.99 | **<0.001** | 0.98 | 0.98 – 0.99 | **<0.001** | |  |
| Compliance | 0.17 | 0.02 – 1.19 | 0.074 | 0.57 | 0.09 – 3.62 | 0.551 | |  |
| **Random Effects** | | | |  | | |  |  |
| σ^2^ | 3.29 | | | 3.29 | | | |  |
| τ_00_ _participant_id:tp_ | 1.25 | | | 0.97 | | | |  |
| τ_00_ _participant_id_ | 0.98 | | | 1.08 | | | |  |
| ICC | 0.44 | | | 0.44 | | | |  |
| Observations | 4069 | | | 4110 | | | |  |
| Marginal R^2^ / Conditional R^2^ | 0.09 / 0.49 | | | 0.07 / 0.48 | | | |  |

**Notes:** Results of the two hierarchical generalized linear models, as implemented in R statistical software, with food habits (model 1) and hygiene habits (model 2) as outcomes. Time point (AN_TP1, AN_TP2) was replaced by Delta BMI-SDS in comparison to the original models. Results report odds ratios, confidence intervals, and p values. AIC/BIC for the adapted models were lower compared to the main models (Food intake AIC=4316 vs. 4310; BIC=4380 vs. 4373; Hygiene AIC= 4316 vs. 4313; BIC= 4379 vs. 4377).

**Table S4: Linear regression predicting Delta-BMI-SDS (weight restoration) with eating habits (robust linear regression and without extreme values)**

| **A Outcome: Delta BMI-SDS** | | | |  | **B Outcome: Delta BMI-SDS** | | | |
| --- | --- | --- | --- | --- | --- | --- | --- | --- |
| *Predictors* | *Estimates* | *95% CI* | *p* |  | *Predictors* | *Estimates* | *95% CI* | *p* |
| (Intercept) | 1.38 | -0.04 – 2.81 | 0.057 |  | (Intercept) | 1.28 | -0.01 – 2.57 | 0.052 |
| Delta Food | -0.87 | -1.50 – -0.24 | **0.008** |  | Delta Food | -0.72 | -1.35 – -0.09 | **0.025** |
| Frequency-Food at AN_TP1 | -0.71 | -1.40 – -0.02 | **0.044** |  | Frequency-Food at AN_TP1 | -0.68 | -1.30 – -0.07 | **0.03** |
| Age | -0.06 | -0.15 – 0.03 | 0.163 |  | Age | -0.05 | -0.13 – 0.03 | 0.223 |
| BMI-SDS at AN_TP1 | -0.75 | -0.88 – -0.61 | **<0.001** |  | BMI-SDS at AN_TP1 | -0.72 | -0.85 – -0.60 | **<0.001** |
| Observations | 44 | | |  | Observations | 43 | | |
| R^2^ / R^2^ adjusted |  |  |  |  | R^2^ / R^2^ adjusted | 0.804 / 0.783 | | |
| **Notes:** Robust linear regression | | | |  | **Notes:** Standard linear regression without the outlier | | | |

**Table S5: Linear regression predicting Delta-BMI-SDS with hygiene habits**

| **Outcome: Delta BMI-SDS** | | | |
| --- | --- | --- | --- |
| *Predictors* | *Estimates* | *95% CI* | *p* |
| (Intercept) | 1.26 | -0.05 – 2.57 | 0.058 |
| Delta Hygiene | -0.49 | -1.11 – 0.14 | 0.121 |
| Frequency-hygiene at AN_TP1 | -0.58 | -1.21 – 0.06 | 0.076 |
| Age | -0.07 | -0.15 – 0.02 | 0.115 |
| BMI-SDS at AN_TP1 | -0.80 | -0.93 – -0.67 | **<0.001** |
| Observations | 44 | | |
| R^2^ / R^2^ adjusted | 0.818 / 0.799 | | |

**Notes:** Linear regression predicting Delta-BMI-SDS with Frequency-hygiene at AN_TP2
